# Normalized lactate load as an independent prognostic indicator in patients with cardiogenic shock

**DOI:** 10.1186/s12872-024-04013-8

**Published:** 2024-07-10

**Authors:** Xia Wu, Lin Yuan, Jiarui Xu, Jing Qi, Keyang Zheng

**Affiliations:** 1 Emergency Critical Care Center, Beijing Anzhen Hospital, Capital Medical University, No.2 Anzhen Road, Chaoyang District, Beijing, 100029 China; 2 Centre of Hypertension, Beijing Anzhen Hospital, Capital Medical University, Beijing, 100029 China

**Keywords:** Lactate, Normalized lactate load, Cardiogenic shock, In-hospital mortality, MIMIC-IV database

## Abstract

**Background:**

Early prognosis evaluation is crucial for decision-making in cardiogenic shock (CS) patients. Dynamic lactate assessment, for example, normalized lactate load, has been a better prognosis predictor than single lactate value in septic shock. Our objective was to investigate the correlation between normalized lactate load and in-hospital mortality in patients with CS.

**Methods:**

Data were extracted from the Medical Information Mart for Intensive Care (MIMIC)-IV database. The calculation of lactate load involved the determination of the cumulative area under the lactate curve, while normalized lactate load was computed by dividing the lactate load by the corresponding period. Receiver Operating Characteristic (ROC) curves were constructed, and the evaluation of areas under the curves (AUC) for various parameters was performed using the DeLong test.

**Results:**

Our study involved a cohort of 1932 CS patients, with 687 individuals (36.1%) experiencing mortality during their hospitalization. The AUC for normalized lactate load demonstrated significant superiority compared to the first lactate (0.675 vs. 0.646, *P* < 0.001), maximum lactate (0.675 vs. 0.651, *P* < 0.001), and mean lactate (0.675 vs. 0.669, *P* = 0.003). Notably, the AUC for normalized lactate load showed comparability to that of the Sequential Organ Failure Assessment (SOFA) score (0.675 vs. 0.695, *P* = 0.175).

**Conclusion:**

The normalized lactate load was an independently associated with the in-hospital mortality among CS patients.

**Supplementary Information:**

The online version contains supplementary material available at 10.1186/s12872-024-04013-8.

## Background

Cardiogenic shock (CS) is a profound cardiac dysfunction leading to diminished cardiac output, critical organ hypoperfusion, and tissue hypoxia. Despite significant advancements in intensive care medicine, high mortality rates in CS, ranging from 40 to 50%, still exist [1]. Arterial lactate, in conditions of oxygen deficiency, is typically elevated in CS. Baseline lactate has been widely used in mortality prediction in CS for decades [2, 3]. Klemm et al. evaluated the efficacy of various lactate measurements within the first 24 h post-ICU admission for predicting 30-day mortality in cardiogenic shock patients, concluding that the 24-hour lactate level is the most effective predictor of mortality in this context [4]. In patients with CS receiving mechanical circulatory support, Scolari et al. found that serum lactate levels and particularly the lactate level after 24 h of treatment were identified as significant independent predictors of 30-day mortality [5]. However, the prognostic accuracy of critically ill patients’ prognosis, as indicated by lactate levels over time, has been shown to be superior with dynamic lactate compared to a single lactate measurement [6, 7].

There are numerous indicators of dynamic lactate changes. Lactate clearance (LC), characterized by the change in lactate levels over time, has been reported with better predictive performance compared to baseline lactate [8]. Another study demonstrated that calculating the weighted average of lactate levels and their changes during the initial 24 h provided superior performance compared to individual measures such as admission, maximum, and minimum lactate levels [7]. Both the lactate value and timing significantly impact clinical outcomes [9]. The concept of normalized lactate load, originally introduced by Zhang et al., served as a comprehensive index that took into account both the lactate value and time [10]. Previous studies have indicated its association with favorable outcomes in septic shock [11] as well as non- septic patients [12]. Nevertheless, the association between this variable and overall mortality has not been validated in a substantial cohort of patients with CS. Therefore, the primary objective of this study was to explore the relationship between normalized lactate load and in-hospital mortality in individuals diagnosed with CS.

## Methods

### Study design

**Fig. 1:**
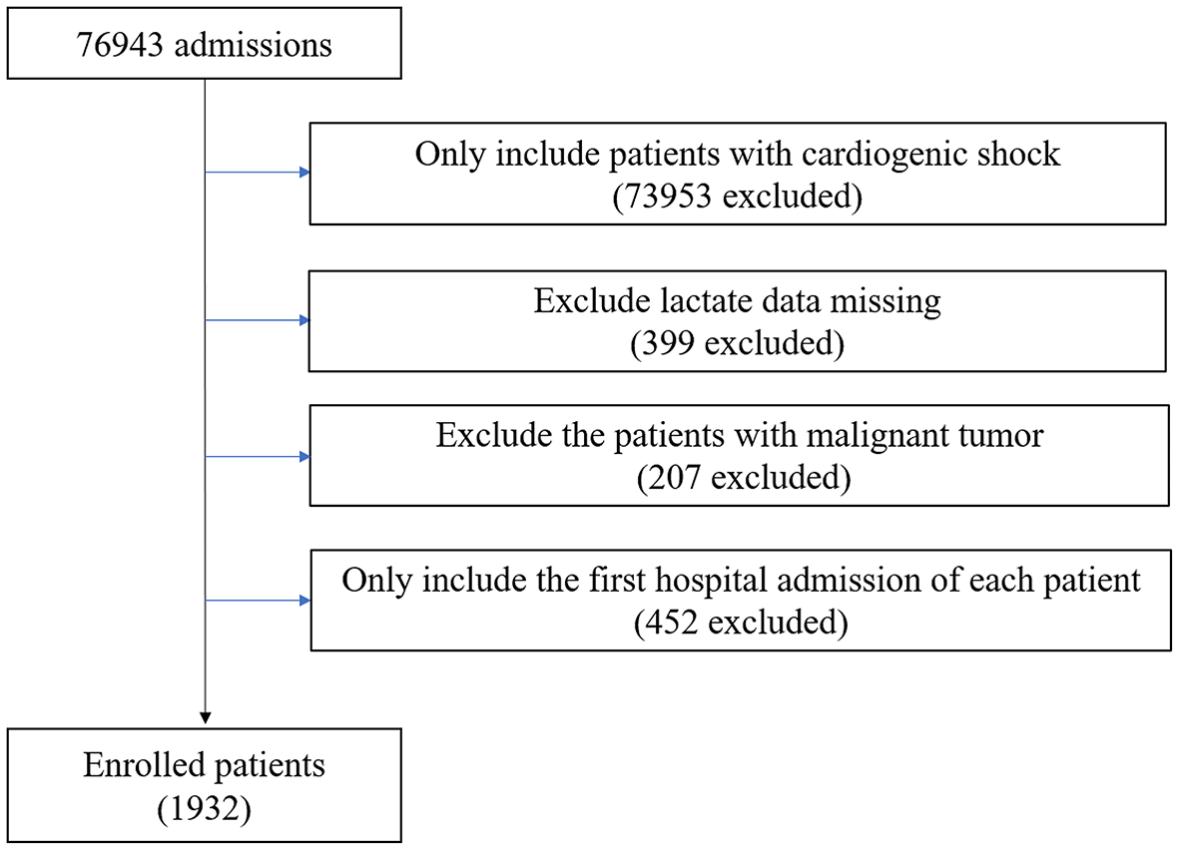
Flow chart of exclusion and inclusion criteria for selecting participants

A retrospective observational study was conducted, including patients diagnosed with CS using the ICD-9/10 code (details can be found in the Supplementary material) [13]. Patients with missing lactate data or those with malignant tumors were excluded from the study. The analysis was exclusively focused on patients initially admitted to the hospital (Fig. 1).

### Data extraction

**Fig. 2:**
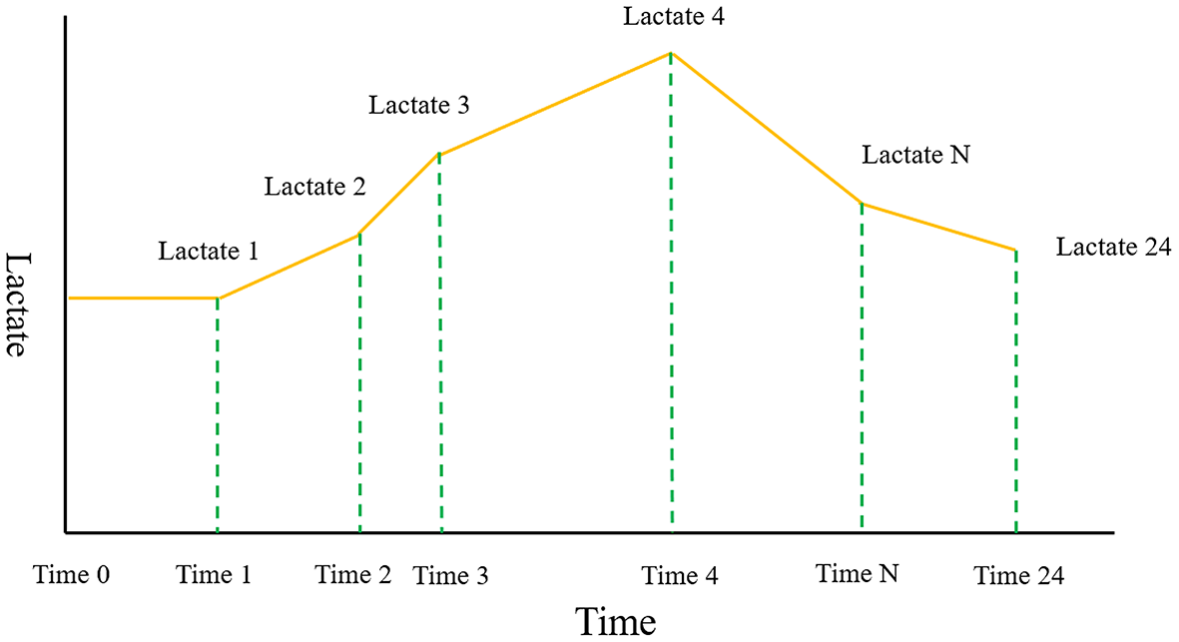
A Cartesian coordinate describing the calculation of Lactate load and normalized Lactate load. “Lactate” represents each Lactate value. “Time” represented their corresponding measurement timestamps. The integral of the curve was computed to quantify the lactate load. The formula for the lactate load was: lactate load=(Lactate_1_ × ΔTime_1_)+(Lactate_2_ × ΔTime_2_)+…+(Lactate_n_ × ΔTime_n_), where Lactate_n_ was the lactate concentration at the nth measurement, and ΔTime_𝑛_ was the time interval since the previous measurement. The normalized lactate load was obtained using the following formula: normalized lactate load = lactate load/24 h

The data used in this study were sourced from the Medical Information Mart for Intensive Care IV (MIMIC-IV, version 2.1) database (Details can be found in Supplementary) [13]. We specifically gathered all lactate measurements and their corresponding measurement timestamps acquired within the initial 24 h following ICU admission to calculate various lactate-related variables, such as normalized lactate load, lactate load, first lactate, maximum lactate, and mean lactate. A Cartesian coordinate graph representing lactate measurements alongside their corresponding timestamps was constructed. The lactate load was computed as the cumulative area under the curve (AUC) of lactate concentration over time. This involved integrating the AUC formed by plotting lactate concentration (mmol/L) against time (hours) from the initial 24 h following ICU admission. The formula for the lactate load was: Lactate Load=(Lactate_1_ × ΔTime_1_)+(Lactate_2_ × ΔTime_2_)+…+(Lactate_n_ × ΔTime_n_), where Lactate_n_ was the lactate concentration at the nth measurement, and ΔTime_𝑛_ was the time interval since the previous measurement. To normalize the lactate load over the total duration of the 24-hour observation period, we divided the total lactate load by 24 h. The formula for normalized lactate load was: Normalized Lactate Load = lactate load/24 h (Fig. 2) [12].

### Grouping and outcome

Participants were stratified into four groups according to quartiles of normalized lactate load. The primary outcome was in-hospital mortality.

### Statistical analysis

Quantitative data with a normal distribution were presented as means and standard deviations (SD), skewed data as medians and interquartile ranges, and categorical data as numbers and percentages. Patient characteristics were compared between groups utilizing appropriate statistical tests such as analysis of variance, Kruskal-Wallis test, and Chi-square test.

A thorough investigation into the correlation between normalized lactate load levels and in-hospital mortality was conducted. This included binary logistic regression analysis using three distinct models (model 1, 2, and 3), where covariates for model 3 were selected through a stepwise method with a removal criterion of *P* > 0.05. The results were presented as odds ratios (OR) with 95% confidence intervals (CI). Furthermore, a restricted cubic spline curve (RCS) was constructed based on model 3, incorporating three knots for analysis.

Receiver operating characteristic (ROC) curves were generated to assess the predictive performance of various lactate parameters, and the area under the curve (AUC) values were compared using the DeLong test.

Subgroup analyses were performed to investigate the associations between normalized lactate load levels and in-hospital mortality across different patient subpopulations.

All analyses, including two-tailed statistical tests with a significance level of *P* < 0.05, were performed using the R software.

## Results

### Patient characteristics

The study included a total of 1,932 CS patients. Participants were stratified into four groups according to quartiles of normalized lactate load: Quartile 1 with normalized lactate load < 1.53 mmol/L (*n* = 483), Quartile 2 with 1.53 mmol/L ≤ normalized lactate load < 2.20 mmol/L (*n* = 483), Quartile 3 with 2.20 mmol/L ≤ normalized lactate load < 3.67 mmol/L (*n* = 483), and Quartile 4 with normalized lactate load ≥ 3.67 mmol/L (*n* = 483). Patients with higher normalized lactate load levels exhibited distinct characteristics (Table 1): they were less likely to be of white ethnicity, and had higher heart rates, while their lower systolic blood pressure was lower. Furthermore, patients in the higher quartiles had elevated levels of white blood cells, neutrophil percentage, hemoglobin, glucose, and sodium, while demonstrating lower platelet levels. They had a higher risk of hypertension and respiratory failure, but a lower risk of congestive heart failure and cardiomyopathy. Additionally, these patients received higher proportions of epinephrine, dialysis, mechanical ventilation, and extracorporeal membrane oxygenation (ECMO) treatment. Notably, these patients also had higher levels of normalized lactate load, lactate load, first lactate, maximum lactate, mean lactate, and Sequential Organ Failure Assessment (SOFA) score.

**Table 1:** Characteristics of patients stratified by normalized lactate load quartiles

Characteristics	Overall (*n* = 1932)	Quartiles of Normalized lactate load				*P* Value
		Quartile1 (*n* = 483)	Quartile2 (*n* = 483)	Quartile3 (*n* = 483)	Quartile4 (*n* = 483)	
Age(years)	67.5 ± 14.5	67.2 ± 14.2	66.7 ± 15.2	68.6 ± 14.1	67.4 ± 14.6	0.224
Sex, n (%)						0.296
Male	1187 (61.4)	287 (59.4)	306 (63.4)	308 (63.8)	286 (59.2)	
Female	745 (38.6)	196 (40.6)	177 (36.6)	175 (36.2)	197 (40.8)	
Ethnicity, n (%)						0.039
White	1229 (63.6)	331 (68.5)	307 (63.6)	320 (66.3)	271 (56.1)	
Black	202 (10.5)	47 (9.7)	55 (11.4)	43 (8.9)	57 (11.8)	
Latino	51 (2.6)	11 (2.3)	10 (2.1)	11 (2.3)	19 (3.9)	
Asian	55 (2.8)	11 (2.3)	15 (3.1)	14 (2.9)	15 (3.1)	
Others	395 (20.4)	83 (17.2)	96 (19.9)	95 (19.7)	121 (25.1)	
Vital signs						
Heart rate (beats/min)	91.8 ± 20.9	88.6 ± 20.2	91.6 ± 20.4	93.5 ± 21.7	93.3 ± 21.1	0.001
Systolic blood pressure (mmHg)	109.1 ± 21.6	108.9 ± 20.9	111.2 ± 21.5	109.7 ± 20.4	106.5 ± 23.2	0.007
Diastolic blood pressure (mmHg)	63.7 ± 18.1	62.8 ± 16.3	65.1 ± 18.4	64.4 ± 17.9	62.3 ± 19.7	0.051
BMI (kg/m^2^)	28.6 ± 7.3	29.1 ± 7.4	28.4 ± 7.6	28.4 ± 7.1	28.5 ± 6.7	0.399
Laboratory parameters						
White blood cell (10^9^/L)	12.7 ± 6.4	11.6 ± 5.5	12.3 ± 5.6	13.0 ± 6.6	13.9 ± 7.4	< 0.001
Neutrophil (%)	80.1 ± 9.9	79.5 ± 9.8	80.6 ± 8.9	80.8 ± 9.6	79.3 ± 11.0	0.043
Lymphocyte (%)	10.8 ± 7.9	11.4 ± 8.2	10.8 ± 7.6	10.3 ± 7.5	10.8 ± 8.0	0.200
Hemoglobin (g/dL)	11.0 ± 2.4	10.8 ± 2.3	11.1 ± 2.3	11.2 ± 2.4	10.9 ± 2.5	0.012
Platelet (10^9^/L)	206.2 ± 99.7	217.4 ± 93.8	211.8 ± 100.0	209.8 ± 107.9	185.7 ± 93.9	< 0.001
Creatinine (mg/dL)	2.0 ± 1.5	2.0 ± 1.6	1.9 ± 1.4	1.9 ± 1.5	2.1 ± 1.6	0.219
Glucose (mg/dL)	169.6 ± 97.8	144.6 ± 64.9	161.1 ± 74.3	165.9 ± 83.9	206.7 ± 139.4	< 0.001
Sodium (mmol/L)	137.5 ± 5.5	137.0 ± 5.3	137.4 ± 5.0	137.1 ± 5.2	138.6 ± 6.2	< 0.001
Potassium (mmol/L)	4.4 ± 0.9	4.4 ± 0.8	4.4 ± 0.9	4.4 ± 0.9	4.4 ± 0.9	0.515
Diagnoses and comorbidities, n (%)						
Hypertension	493 (25.5)	111 (23.0)	117 (24.2)	114 (23.6)	151 (31.3)	0.010
Diabetes	773 (40.0)	186 (38.5)	199 (41.2)	178 (36.9)	210 (43.5)	0.160
Dyslipidemia	989 (51.2)	245 (50.7)	249 (51.6)	254 (52.6)	241 (49.9)	0.857
Cerebrovascular disease	233 (12.1)	61 (12.6)	49 (10.1)	58 (12.0)	65 (13.5)	0.439
Congestive heart failure	1539 (79.7)	414 (85.7)	408 (84.5)	406 (84.1)	311 (64.4)	< 0.001
Coronary artery disease	1373 (71.1)	342 (70.8)	352 (72.9)	342 (70.8)	337 (69.8)	0.754
Myocardial infarction	945 (48.9)	241 (49.9)	251 (52.0)	224 (46.4)	229 (47.4)	0.300
Myocarditis	16 (0.8)	4 (0.8)	2 (0.4)	5 (1.0)	5 (1.0)	0.679
Atrial fibrillation	1232 (63.8)	299 (61.9)	311 (64.4)	323 (66.9)	299 (61.9)	0.315
Cardiomyopathy	393 (20.3)	96 (19.9)	131 (27.1)	98 (20.3)	68 (14.1)	< 0.001
Respiratory failure	1011 (52.3)	231 (47.8)	249 (51.6)	247 (51.1)	284 (58.8)	0.006
Chronic renal disease	782 (40.5)	208 (43.1)	202 (41.8)	196 (40.6)	176 (36.4)	0.174
Treatment, n (%)						
Dobutamine	537 (27.8)	114 (23.6)	145 (30.0)	142 (29.4)	136 (28.2)	0.108
Dopamine	439 (22.7)	113 (23.4)	104 (21.5)	96 (19.9)	126 (26.1)	0.119
Epinephrine	585 (30.3)	63 (13.0)	101 (20.9)	160 (33.1)	261 (54.0)	< 0.001
Corticosteroids	590 (30.5)	160 (33.1)	139 (28.8)	148 (30.6)	143 (29.6)	0.488
Dialysis	408 (21.1)	86 (17.8)	83 (17.2)	98 (20.3)	141 (29.2)	< 0.001
Mechanical vent	1216 (62.9)	259 (53.6)	284 (58.8)	295 (61.1)	378 (78.3)	< 0.001
ECMO	81 (4.2)	7 (1.4)	18 (3.7)	11 (2.3)	45 (9.3)	< 0.001
Revascularization therapy	471 (24.4)	112 (23.2)	114 (23.6)	128 (26.5)	117 (24.2)	< 0.001
IABP	138 (7.1)	39 (8.1)	38 (7.9)	28 (5.8)	33 (6.8)	0.493
IMPELLA	107 (5.5)	22 (4.6)	25 (5.2)	20 (4.1)	20 (8.3)	0.021
Normalized lactate load (mmol/L)	3.1 ± 2.5	1.2 ± 0.2	1.9 ± 0.2	2.8 ± 0.4	6.6 ± 2.8	< 0.001
Lactate load (mmol·hr/L)	74.7 ± 60.4	29.1 ± 5.4	44.7 ± 4.4	67.0 ± 10.1	158.1 ± 66.6	< 0.001
First lactate (mmol/L)	3.3 ± 2.7	1.4 ± 0.5	2.2 ± 0.8	3.3 ± 1.5	6.3 ± 3.4	< 0.001
Maximum lactate (mmol/L)	4.3 ± 3.4	1.5 ± 0.5	2.5 ± 0.8	4.2 ± 1.6	9.0 ± 3.1	< 0.001
Mean lactate (mmol/L)	3.2 ± 2.3	1.3 ± 0.3	2.0 ± 0.3	3.0 ± 0.7	6.7 ± 2.6	< 0.001
SOFA	9 [6, 12]	7 [5, 10]	8 [5, 11]	9 [6, 12]	12 [9, 14]	< 0.001
In-hospital mortality, n (%)	697 (36.1)	117 (24.2)	131 (27.1)	146 (30.2)	303 (62.7)	< 0.001

Continuous variables were presented as mean ± SD or median. Categorical variables were presented as number (percentage). Abbreviation: BMI: body mass index; SOFA: sequential organ failure assessment; ECMO: extracorporeal membrane oxygenation; IABP: intra-aortic balloon pump

### Association between normalized lactate load and in-hospital mortality

The overall in-hospital mortality rate was 36.1%. A significant trend was observed, indicating a higher in-hospital mortality with increasing normalized lactate load (quartile 4 vs. quartile 1: 62.7% vs. 24.2%, *P* < 0.001) as shown in Table 1. Binary logistic regression analysis was performed to confirm the direct impact of normalized lactate load on in-hospital mortality. In Model 1, a significant association between elevated levels of normalized lactate load and increased risk of in-hospital mortality was observed (quartile 4 vs. quartile 1: OR, 95% CI: 5.27, 4.00–6.97, *P* < 0.001, P for trend < 0.001). Model 2 revealed a positive correlation between normalized lactate load and mortality (quartile 4 vs. quartile 1: OR, 95% CI: 5.39, 4.07–7.16, *P* < 0.001, P for trend < 0.001). Even after adjusting for additional confounding variables in Model 3, a significant association persisted between higher quartiles of normalized lactate load and an increased risk of in-hospital mortality (quartile 4 vs. quartile 1: OR, 95% CI: 3.56, 2.52–5.06, *P* < 0.001, P for trend < 0.001). Furthermore, when considering normalized lactate load as a continuous variable, each unit increase was associated with approximately 0.72-fold, 0.73-fold, and 0.47-fold increases in the risk of in-hospital mortality in Model 1, Model 2, and Model 3, respectively (Table 2).

**Table 2:** The association between normalized lactate load and in-hospital mortality

	OR (95% CI)	*P* Value
Model 1		
Quartile 1	Reference	
Quartile 2	1.16 [0.87, 1.56]	0.303
Quartile 3	1.36 [1.02, 1.80]	0.036
Quartile 4	5.27 [4.00, 6.97]	< 0.001
Continuous	1.72 [1.57, 1.88]	< 0.001
Model 2		
Quartile 1	Reference	
Quartile 2	1.18 [0.88, 1.58]	0.264
Quartile 3	1.37 [1.03, 1.83]	0.031
Quartile 4	5.39 [4.07, 7.16]	< 0.001
Continuous	1.73 [1.58, 1.89]	< 0.001
Model 3		
Quartile 1	Reference	
Quartile 2	1.15 [0.83, 1.59]	0.414
Quartile 3	1.13 [0.82, 1.57]	0.452
Quartile 4	3.56 [2.52, 5.06]	< 0.001
Continuous	1.47 [1.31, 1.64]	< 0.001

Models were derived from binary m. Model 1: unadjusted. Model 2: adjusted for age, gender, ethnicity. Model 3: adjusted for age, gender, ethnicity, heart rate, systolic blood pressure, white blood cell, hemoglobin, sodium, SOFA, hypertension, congestive heart failure, myocardial infarction, dyslipidemia, respiratory failure, chronic renal disease, dobutamine, dopamine, epinephrine, corticosteroids, dialysis, ECMO. Abbreviation: OR: odds ratio; CI: confidence interval; ECMO: extracorporeal membrane oxygenation; SOFA: sequential organ failure

**Fig. 3:**
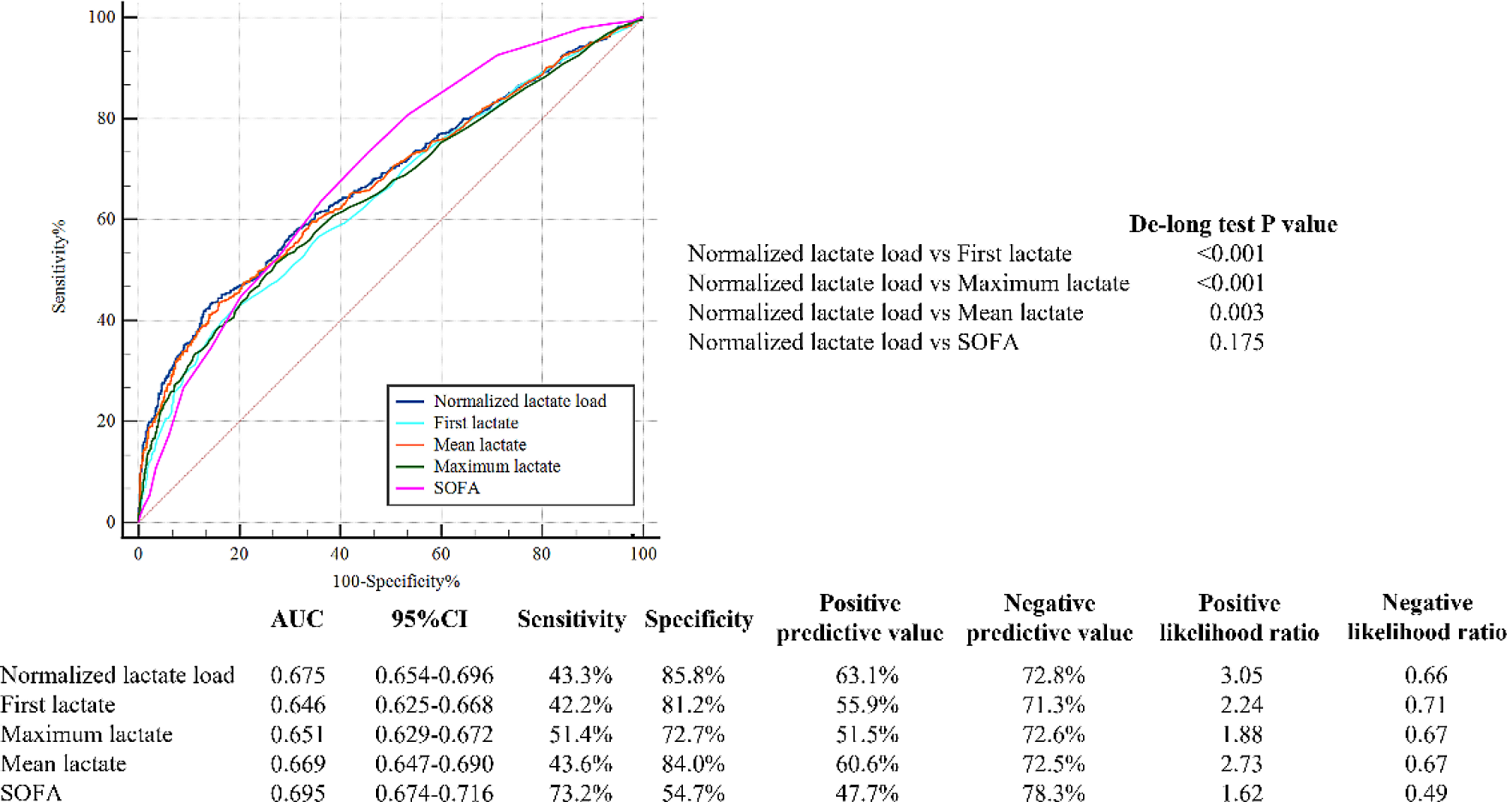
ROC curves for the prediction of in-hospital mortality of normalized lactate load, first lactate, maximum lactate, mean lactate, SOFA. Abbreviation: ROC: receiver operating characteristic; SOFA: sequential organ failure

In Fig. 3, the ROC curves visually depicted the predictive capability of normalized lactate load for in-hospital mortality, yielding an AUC of 0.675 [95% CI: 0.654–0.696]. This performance surpassed that of the first lactate (0.675 vs. 0.646, De-long test *P* < 0.001), maximum lactate (0.675 vs. 0.651, De-long test *P* < 0.001), and mean lactate (0.675 vs. 0.669, De-long test *P* = 0.003). Notably, it demonstrated comparability to the predictive performance of SOFA score (0.675 vs. 0.695, De-long test *P* = 0.175).

**Fig. 4:**
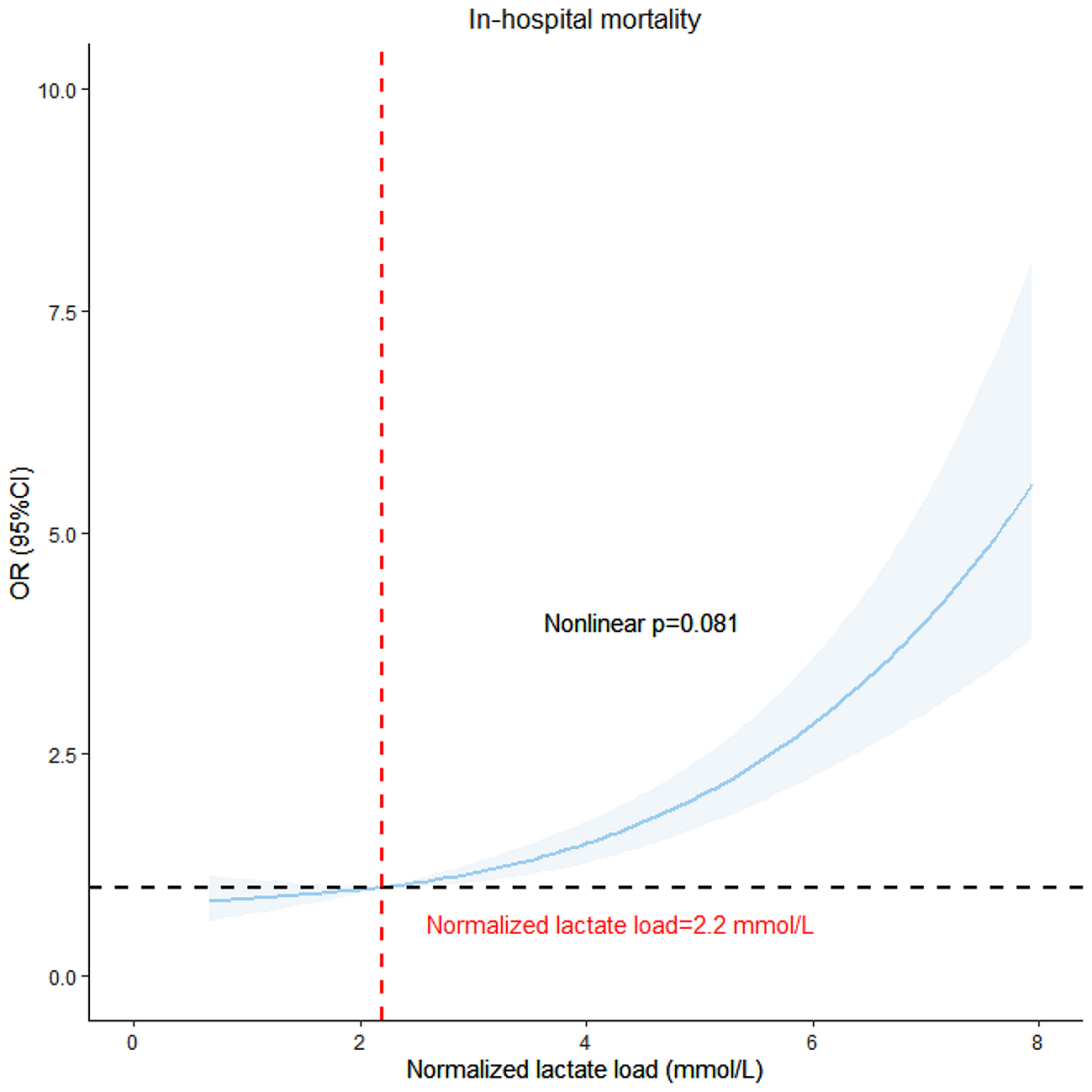
RCS model showing the association between normalized lactate load and in-hospital mortality. Abbreviation: RCS: restricted cubic spline curve

In Fig. 4, the association between in-hospital mortality and normalized lactate load was visualized using RCS with model 3 applied. Upon adjusting for potential confounding factors, an evident linear relationship between normalized lactate load and in-hospital mortality was identified, with a non-linear p-value of 0.081.

### Subgroup analysis

In all subgroups, except for those categorized by myocarditis and ethnicity, elevated levels of normalized lactate load were significantly associated with an increased risk of in-hospital mortality. Significant interactions were notably detected among subgroups including septic shock, heart rate, platelets, cerebrovascular disease, atrial fibrillation, diabetes, and respiratory failure (Table can be found in the Supplementary material).

## Discussion

Our study indicated a significant correlation between normalized lactate load and in-hospital mortality in patients with CS. Increased normalized lactate load was associated with an elevated risk of in-hospital death, even after adjusting for potential confounders. Notably, normalized lactate load exhibited superior predictive ability for in-hospital mortality compared to the first lactate value, maximum lactate value, and mean lactate value, and was comparable to the SOFA score.

In patients with CS, reduced cardiac output leads to inadequate tissue perfusion and decreased oxygen consumption, further resulting in increased lactate levels [14]. Elevated lactate levels in CS are associated with adverse outcomes due to a complex interplay of factors. The primary driver is tissue hypoperfusion from reduced cardiac output, which shifts metabolism towards anaerobic glycolysis, escalating lactate production. Impaired lactate clearance, often due to hepatic dysfunction, further exacerbates this accumulation. Additionally, adrenergic stimulation during shock increases glycolysis, potentially overwhelming the tricarboxylic acid cycle’s capacity to metabolize pyruvate, thereby augmenting lactate concentrations [15, 16]. Numerous studies have shown the importance of lactate in predicting the outcome of critically ill patients [17, 18]. In a recent study, baseline lactate value was incorporated in the early risk stratification model: IABP-SHOCK II ((Intra-aortic Balloon Pump in Cardiogenic Shock II) score in patients with CS [19]. However, the severity of organ damage is determined by the combination of lactate concentration and duration, and a single lactate value might not be comprehensive. Lactate load, defined as the AUC of lactate concentration over time, representes a composite measure of both lactate concentration and duration. Moreover, normalized lactate load, calculated by dividing AUC by time interval, provides an estimate of average intensity of hyperlactatemia. Chen et al. documented that normalized lactate load emerged as an independent risk factor for 28-day mortality in patients with septic shock, demonstrating a superior prognostic impact compared to baseline and maximum lactate levels [11]. In non-septic shock patients, the predictive ability of normalized lactate load was also superior to a single lactate value, although the accuracy was lower compared to sepsis patients [12]. In critically ill pediatric patients, normalized lactate load was identified as an independent predictor of adverse outcomes [20]. In a prior investigation, a significant association was established between normalized lactate levels and the incidence of acute kidney injury in patients undergoing cardiopulmonary bypass surgery [10]. Our study is the first to find the correlation between normalized lactate load and in-hospital mortality in individuals with CS.

Early assessment of prognosis and initiation of mechanical circulatory was crucial for patients with CS. However, there was a lack of markers to guide early treatment decisions. In patients with CS, lactate levels are elevated due to tissue and organ hypoperfusion. Target organs failure including kidney and liver failure caused by venous congestion, might further contribute to decreased lactate clearance [21, 22]. The prognostic value of a single value of lactate or LC was evaluated in previous study. In a sub-analysis of the IABP-SHOCK II trial and corresponding registry, LC was defined as the exact time difference between admission and 8 h after admission. A lactate level 8 h after admission was superior in mortality prediction compared to admission value or LC [23]. However, in a post hoc analysis of the DOREMI (Dobutamine Compared to Milrinone in the Treatment of Cardiogenic Shock) trial, complete LC was a strong predictor of in-hospital mortality in different time [24]. In our study, lactate load was calculated as the AUC of the lactate, which was more intuitive and could reflect the concentration and duration of lactate. Meanwhile, patients in IABP-SHOCK II and DOREMI trials were highly selected as they were in randomized control trials. Our study conducted subgroup analyses on heterogeneous patients, which may provide more representative results. These findings suggested that normalized lactate load was a robust predictor of mortality, consistent across diverse physiological profiles.

Lactate area, as a similar indicator with lactate load, was also evaluated in previous studies. One observational study calculated lactate area as the sum of the AUC of serial lactate levels measured every 6 h during the 24 h after admission [25]. The lactate area showed more substantial predictive power (AUC = 0.83) than the initial lactate level (AUC = 0.70) and 24 h lactate clearance (AUC = 0.72). In another study, a higher lactate area in patients with septic shock also indicated higher 28-day mortality [26]. However, the lactate area was not comparable when the length of lactate testing differed in different patients. The normalized lactate load, which represented the average lactate load during the same period, was standardized by time to enhance comparability. Our study focused on normalized lactate load, revealing a significant association between an elevated normalized lactate load and increased in-hospital mortality. Furthermore, the predictive efficacy of normalized lactate load proved to be more robust than that of initial lactate, maximum lactate, and mean lactate. This was consistent with previous findings in patients with septic shock [11]. Notably, the widely used SOFA score evaluates the prognosis of critically ill patients, including those with CS, through a complex assessment involving multiple indicators [27–32]. Encouragingly, our study revealed that normalized lactate load exhibited a predictive capability for in-hospital mortality comparable to the traditional prognostic indicator, SOFA score. Furthermore, the calculation of normalized lactate load was simpler compared to SOFA as it relied on a single lactate dimension. These findings imply that in resource-limited primary hospitals, for patients with CS, when obtaining the necessary indicators for calculating SOFA is challenging, normalized lactate load derived from routine laboratory tests can serve as an alternative predictive marker. Its utilization enables the prompt identification of high-risk and high-mortality patients, facilitating timely clinical interventions to enhance patient outcomes.

## Limitation

However, our study faced specific limitations. As a retrospective analysis, determining the specific etiologies of CS for each case proved challenging, with diagnoses based solely on ICD codes rather than formal clinical criteria. This reliance on administrative rather than clinical coding may impact the accuracy and specificity of identifying genuine CS cases. Additionally, excessive missing data for inflammatory markers such as CRP (C-reactive protein) and PCT (procalcitonin) precluded their inclusion in the baseline assessment. The use of the U.S.-specific MIMIC-IV database potentially limits the generalizability of our findings. Moreover, the treatment of nearly 20% of the patients with dialysis could have influenced lactate values, impacting the assessment of normalized lactate load, which although superior to single lactate measurements in predicting mortality, presents negligible absolute differences in AUC, limiting its practical clinical application. The study was also constrained to evaluating only in-hospital mortality. Long-term outcomes and their relationship with normalized lactate load require further investigation. Lastly, while normalized lactate load offers higher predictive value for in-hospital mortality than initial or maximum lactate values, its complex calculation could hinder rapid clinical deployment. Balancing predictive accuracy with operational simplicity is essential, suggesting future research should aim to simplify methodologies to enhance clinical utility.

## Conclusion

In patients with CS, the normalized lactate load has been recognized as an independent predictor of in-hospital mortality, exhibiting a predictive capacity that was comparable to the SOFA score. This underscores the potential value of normalized lactate load as a prognostic marker and its utility in guiding treatment decisions for individuals with CS.

### Electronic supplementary material

Below is the link to the electronic supplementary material.

Supplementary Material 1

## Data Availability

The data used in this study was from the Medical Information Mart for Intensive Care IV (MIMIC-IV, version 2.1) database (https://physionet.org/content/mimiciv/2.2/). The authors were approved to access to the database through (ID:12353225).

## Abbreviations

BMI: Body Mass Index
SOFA: Sequential Organ Failure Assessment
ECMO: Extracorporeal Membrane Oxygenation
OR: Odds Ratio
CI: Confidence Interval
CS: Cardiogenic Shock
MIMIC: Medical Information Mart for Intensive Care
ROC: Receiver Operating Characteristic
AUC: Areas Under The Curves
LC: Lactate Clearance
RCS: Cubic Spline Curve
