# Supplementary material for: Normalized lactate load as an independent prognostic indicator in patients with cardiogenic shock
Source: BMC Cardiovasc Disord. 2024 Jul 10;24:348. doi: 10.1186/s12872-024-04013-8 (PMC11234684; doi:10.1186/s12872-024-04013-8)
Supplement: Supplementary file 1 — Supplementary Material 1 [file 12872_2024_4013_MOESM1_ESM.docx]

**Cardiogenic shock ICD-9/10 Code.**

78551

R570

T8111

T8111XA

T8111XS

T8111XD

MIMIC-IV.

The data used in this study were sourced from the Medical Information Mart for Intensive Care IV (MIMIC-IV, version 2.1) database. Renowned for its comprehensive and high-quality data, this database captures information on patients admitted to intensive care units (ICUs) at the Beth Israel Deaconess Medical Center from 2008 to 2019

**Data extraction.**

Following data were collected: demographics (age, sex, ethnicity), vital signs (heart rate, systolic blood pressure, diastolic blood pressure), BMI, laboratory parameter (white blood cell, neutrophil (%), lymphocyte (%), hemoglobin, platelet, creatinine, glucose, sodium, potassium), diagnoses and comorbidities (hypertension, diabetes, dyslipidemia, cerebrovascular disease, congestive heart failure, coronary artery disease, myocardial infarction, myocarditis, atrial fibrillation, cardiomyopathy, respiratory failure, chronic renal disease), treatment(dobutamine, dopamine, epinephrine, corticosteroids, dialysis, mechanical vent, ECMO, revascularization therapy, IABP, IMPELLA), normalized lactate load, lactate load, first lactate, maximum lactate, mean lactate, SOFA.

Abbreviation: BMI: body mass index; ECMO: extracorporeal membrane oxygenation; SOFA: sequential organ failure assessment; IABP: intra-aortic balloon pump.

Supplementary Table. Subgroup analysis

| Subgroup | N | OR (CI) | P  for interaction |
| --- | --- | --- | --- |
| Age (years) |  |  | 0.753 |
| <70 | 996 | 1.39(1.31-1.49) |  |
| >=70 | 936 | 1.42(1.32-1.53) |  |
| Sex |  |  | 0.744 |
| Male | 1187 | 1.41(1.32-1.50) |  |
| Female | 745 | 1.38(1.28-1.49) |  |
| Ethnicity |  |  | 0.365 |
| White | 1229 | 1.42(1.33-1.52) |  |
| Black | 202 | 1.32(1.18-1.48) |  |
| Latino | 51 | 1.43(1.10-1.86) |  |
| Asian | 55 | 1.17(0.93-1.46) |  |
| Others | 395 | 1.46(1.31-1.47) |  |
| AST (U/L) |  |  | 0.091 |
| <69 | 897 | 1.27 (1.17-1.37) |  |
| >=69 | 905 | 1.39(1.30-1.48) |  |
| ALT (U/L) |  |  | 0.106 |
| <43 | 887 | 1.29(1.20-1.39) |  |
| >=43 | 913 | 1.41(1.31-1.50) |  |
| Respiratory rate (beats/min) |  |  | 0.516 |
| <20 | 905 | 1.36(1.27-1.47) |  |
| >=20 | 1026 | 1.41(1.32-1.51) |  |
| Heart rate (beats/min) |  |  | 0.033 |
| <89 | 943 | 1.48(1.37-1.59) |  |
| >=89 | 989 | 1.33(1.25-1.42) |  |
| Platelet (10^9^/L) |  |  | 0.004 |
| <192 | 956 | 1.31(1.24-1.40) |  |
| >=192 | 976 | 1.52(1.40-1.65) |  |
| Sodium (mmol/L) |  |  | 0.082 |
| <138 | 914 | 1.33(1.24-1.44) |  |
| >=138 | 1018 | 1.46(1.37-1.56) |  |
| Chloride (mmol/L) |  |  | 0.349 |
| <101 | 849 | 1.36(1.26-1.46) |  |
| >=101 | 1079 | 1.42(1.33-1.52) |  |
| Glasgow Coma Scale |  |  | 0.670 |
| <15 | 286 | 1.23(1.11-1.36) |  |
| =15 | 1631 | 1.43(1.36-1.52) |  |
| IABP |  |  |  |
| Yes | 138 | 1.46(1.19-1.79) |  |
| No | 1794 | 1.39(1.33-1.46) |  |
| IMPELLA |  |  | 0.957 |
| Yes | 107 | 1.40(1.15-1.70) |  |
| No | 1825 | 1.39(1.33-1.46) |  |
| Congestive heart failure |  |  | 0.896 |
| Yes | 1539 | 1.38(1.30-1.46) |  |
| No | 393 | 1.39(1.26-1.52) |  |
| Coronary artery disease |  |  | 0.828 |
| Yes | 1373 | 1.41(1.33-1.49) |  |
| No | 559 | 1.39(1.27-1.52) |  |
| Myocardial infarction |  |  | 0.201 |
| Yes | 945 | 1.45(1.35-1.56) |  |
| No | 987 | 1.36(1.27-1.45) |  |
| Myocarditis |  |  | 0.715 |
| Yes | 16 | 1.52(0.97-2.37) |  |
| No | 1916 | 1.40(1.33-1.47) |  |
| Cerebrovascular disease |  |  | 0.004 |
| Yes | 233 | 1.20(1.08-1.34) |  |
| No | 1699 | 1.44(1.36-1.52) |  |
| Atrial fibrillation |  |  | 0.019 |
| Yes | 1232 | 1.34(1.26-1.42) |  |
| No | 700 | 1.51(1.39-1.64) |  |
| Cardiomyopathy |  |  | 0.973 |
| Yes | 393 | 1.39(1.23-1.57) |  |
| No | 1539 | 1.39(1.32-1.47) |  |
| Hypertension |  |  | 0.788 |
| Yes | 493 | 1.39(1.27-1.52) |  |
| No | 1439 | 1.41(1.33-1.50) |  |
| Diabetes |  |  | 0.031 |
| Yes | 773 | 1.32(1.23-1.41) |  |
| No | 1159 | 1.46(1.37-1.57) |  |
| Hypercholesterolemia |  |  | 0.167 |
| Yes | 989 | 1.45(1.35-1.55) |  |
| No | 943 | 1.35(1.27-1.44) |  |
| Respiratory failure |  |  | <0.001 |
| Yes | 1011 | 1.26(1.19-1.33) |  |
| No | 921 | 1.63(1.50-1.78) |  |
| Chronic kidney disease |  |  | 0.527 |
| Yes | 782 | 1.38(1.28-1.50) |  |
| No | 1150 | 1.43(1.34-1.52) |  |
| Septic shock |  |  | <0.001 |
| Yes | 421 | 1.19(1.09-1.30) |  |
| No | 1511 | 1.48(1.40-1.57) |  |

Abbreviation: OR: odds ratio; CI: confidence; AST: aspartate aminotransferase; ALT: alanine aminotransferase; IABP: intra-aortic balloon pump.
